# Circumferential Compression Encoding (CIRCOME) using Polar K-space

**DOI:** 10.1186/1532-429X-18-S1-P327

**Published:** 2016-01-27

**Authors:** Shokoufeh Golshani, Abbas Nasiraei-Moghaddam

**Affiliations:** Amirkabir University of Technology, Tehran, Iran (the Islamic Republic of)

## Background

Circumferential strain of the Left Ventricle gives valuable insights into regional myocardial function. This strain can be calculated from the density of radial taglines estimated by CIRCOME method [1], which benefits from direct use of tagline information in the frequency domain. CIRCOME exploits circular bandpass filters in order to selectively separate various frequencies in this region and reconstruct compression encoded images. A series of 2D filtering over a Cartesian grid is computationally expensive and also results in round-off errors. Therefore, direct extraction of the effective frequencies in k-space has a potential advantage of producing density maps faster and with higher accuracy. The purpose of this study was to investigate the feasibility of an efficient polar approach for CIRCOME method applicable on radial data acquisition in combination with an adapted Polar Fourier Transform [2].

## Methods

Mid-ventricular short axis myocardial images with radial tag pattern of one healthy volunteer were acquired using a 2D segmented radial k-space trajectory on a 1.5T Siemens TIM Avanto scanner with 88, 64, and 40 radial spokes. All datasets consisted of 19 frames in a full cardiac cycle and took 11.7, 8.5 and 5.3 seconds, respectively. The radial raw data was transposed to generate the circular rings format. Each distinct circle in the annular sub-region of k-space, indicating the specific frequency of tagging modulation, was then individually used through a Hankel-based algorithm, to reconstruct an image. These images were then 2D cross-correlated in the polar coordinate system with the image obtained from the full k-space data and finally the compression encoded density maps were estimated directly in the polar domain.

## Results

Density maps of cardiac frames from in-vivo heart images with 88 radial spokes extracted by the previously-developed CIRCOME algorithm. The corresponding density maps were then calculated by the proposed method for 88-, 64-, and 40-spoke acquisitions. The resulting density maps for the *6*^*th*^ frame are illustrated in Figure 2. The Normalized RMS Errors (NRMSE) between calculated density maps of the proposed and previous CIRCOME methods have been calculated, which are 0.0124, 0.0168, and 0.0215 for density maps obtained from 88-, 64-, and 40-spoke images, respectively. The images show high similarity while the proposed algorithm speeds up both the acquisition and processing of radial taglines more than 2 times. The results also confirm the robustness of the approach against reduced number of spokes which proportionally corresponds to the acceleration of acquisition time.

**Figure 1:**
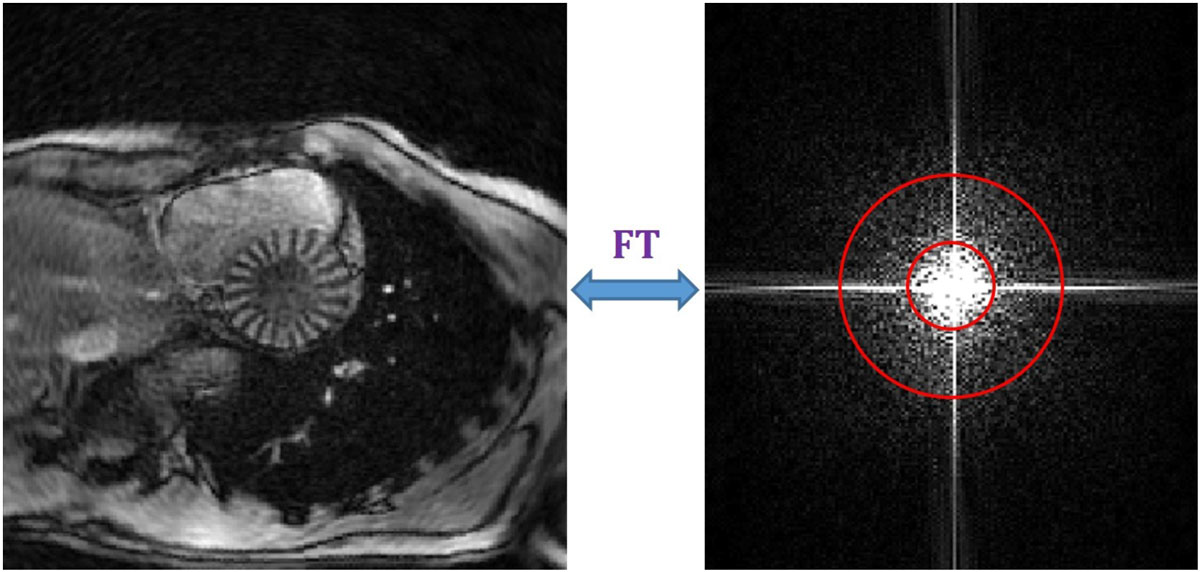
**Tagged reconstructed image of the 6**^**th**^ **cardiac frame by the adapted PFT (left) and its corresponding k-space (right)**. The specified region demonstrates the tagging energy in the K-space.

**Figure 2:**
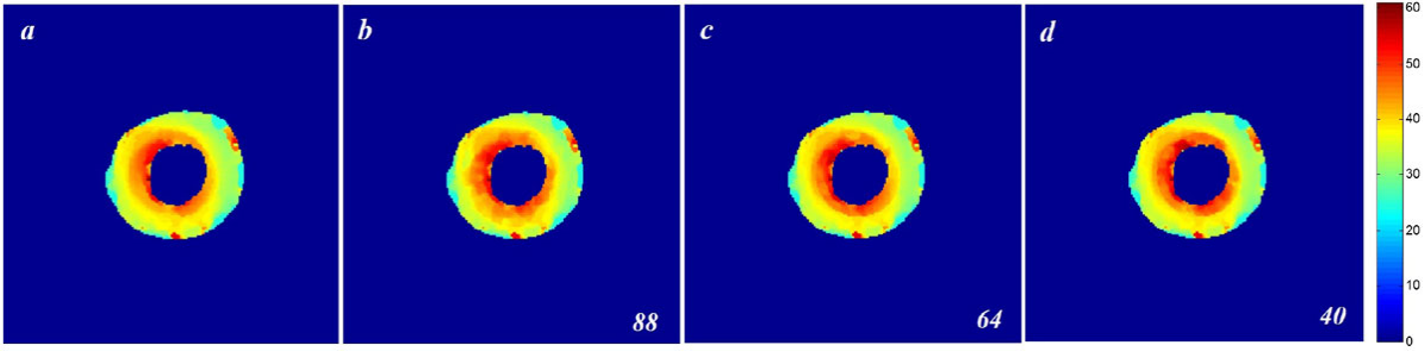
**Radial tagline density maps of the** ***6***^***th***^ **cardiac frames of one volunteer acquired through radial k-space sampling with 88 radial spokes were derived by using (a) Previously-developed CIRCOME method and (b) our proposed method**. (c-d): The corresponding maps from 64 and 40-view images.

## Conclusions

We have shown that the analysis of radially tagged images can become more efficient and simpler if the data is sampled through radial acquisitions. Our proposed approach will provide efficient and accurate myocardial strain assessment with high reliability.

## References

[1] Moghaddam, Finn (2008). SPIE. Med Img Physiology.

[2] Guo, Song (2004). ISMRM.

